# CUT&Tag Applied to Zebrafish Adult Tail Fins Reveals a Return of Embryonic H3K4me3 Patterns During Regeneration

**DOI:** 10.21203/rs.3.rs-4189493/v1

**Published:** 2024-04-03

**Authors:** Phu Duong, Anjelica Rodriguez-Parks, Junsu Kang, Patrick J Murphy

**Affiliations:** University of Rochester; University of Wisconsin-Madison; University of Wisconsin-Madison; University of Rochester

## Abstract

Regenerative potential is governed by a complex process of transcriptional reprogramming, involving chromatin reorganization and dynamics in transcription factor binding patterns throughout the genome. The degree to which chromatin and epigenetic changes contribute to this process remains partially understood. Here we provide a modified CUT&Tag protocol suitable for improved characterization and interrogation of epigenetic changes during adult fin regeneration in zebrafish. Our protocol generates data that recapitulates results from previously published ChIP-Seq methods, requires far fewer cells as input, and significantly improves signal to noise ratios. We deliver high-resolution enrichment maps for H3K4me3 of uninjured and regenerating fin tissues. During regeneration, we find that H3K4me3 levels increase over gene promoters which become transcriptionally active and genes which lose H3K4me3 become silenced. Interestingly, these epigenetic reprogramming events recapitulate the H3K4me3 patterns observed in developing fin folds of 24-hour old zebrafish embryos. Our results indicate that changes in genomic H3K4me3 patterns during fin regeneration occur in a manner consistent with reactivation of developmental programs, demonstrating CUT&Tag to be an effective tool for profiling chromatin landscapes in regenerating tissues.

Epigenetic control of chromatin states defines cellular programming, facilitates response to extrinsic signals, and enables maintenance of cell identity during proliferation. In the context of development, highly regulated epigenetic patterns and changes in cell-specific transcription factor binding patterns form the scaffold upon which gene transcription is regulated (1–3). For instance, tri-methylation of lysine 4 on the tail of histone H3 (H3K4me3) associates with active chromatin regions and promotes RNA polymerase occupancy over genic promoter regions (2, 4). Genomics patterns for these types of transcription-associated histone modifications have been widely established for numerous tissues of *Danio rerio* during embryogenesis and development, but patterns during regeneration remain less well defined (5–7).

During fin regeneration in zebrafish, dramatic cellular events occur over the first few days post-amputation (dpa), including an initial phase of healing, followed by wound epidermis formation, blastema formation, cell proliferation, and redifferentiation (8). Rather than reliance on resident stem cell populations, the regeneration process involves dedifferentiation of adult fin tissues in order to establish heterogenous progenitor cell populations within the blastema (9), occurring at 1–2 dpa. Prior studies have investigated how epigenetic and chromatin modifications support the regeneration process in caudal fins, including studies which identified tissue regeneration-specific enhancers (10), chromatin accessibility changes during regeneration (7), and the importance of removing tri-methylation at 27th lysine of the histone H3 tail (H3K27me3) from many genes (11). Despite these successes, knowledge of epigenetic reprogramming during caudal fin regeneration is much more limited than similar reprogramming processes occurring within embryos (12–16), likely due to challenges associated with genome-wide characterization of epigenetic marks in adult differentiated tissues.

Chromatin immuno-precipitation combined with sequencing (ChIP-Seq) (17) is the standard methodology for profiling histone modifications and has proven to be a useful tool in many systems (18, 19). This method enables high throughput DNA sequencing to map the genomic binding sites of target proteins and provides valuable information for profiling the relative chromatin states of cells (18). However, ChIP-Seq methods typically require a significantly large number of cells (often > 1-million cells per replicate), inhibiting experimentation in many situations. Additionally, biases intrinsic to sonication and chromatin purifications can also cause significant issues with ChIP-Seq, leading to decreased signal-to-noise ratios (20). Recently, a newer method called Cleavage Under Targets and Tagmentation, or CUT&Tag, (21, 22) has been developed which overcomes many of these limitations, and has the potential to allow researchers to interrogate additional tissues or cell types (14, 21). Like ChIP-seq, CUT&Tag is an antibody-based technology that detects protein-DNA interactions, but instead of sonication and crosslinking, CUT&Tag takes advantage of a protein A/G to Tn5 fusion, enabling users to specifically cut and amplify DNA at precise locations where antibodies bind genomic chromatin. This difference provides a significant advantage, decreasing sample loss and significantly reducing sequencing levels over background regions. Here we have developed a modified CUT&Tag protocol, which has enabled us to study the active histone mark H3K4me3 in both intact and regenerating zebrafish caudal fins.

To investigate how epigenetic changes associate with the regeneration process, we applied CUT&Tag to cells isolated from uninjured and regenerating fins. We find that many genes which acquire H3K4me3 during regeneration are known to be involved in the establishment of embryonic morphology, including a large number of loci which possessed high levels of H3K4me3 at 24hpf (hours post fertilization) in embryonic fin folds. Our results support a model in which the regeneration process relies on reactivation of dormant epigenetic programs that are utilized initially during embryogenesis (23), and demonstrate the strong utility of CUT&Tag applied during zebrafish caudal fin regeneration. It is our hope that data from this study will serve as an example for future researchers investigating chromatin changes in adult zebrafish tissues, and provide a resource for subsequent investigation of regeneration.

## Results

### CUT&Tag detects high H3K4me3 levels over gene promoters in caudal fin with strong reproducibility.

**Figure 1 -:**
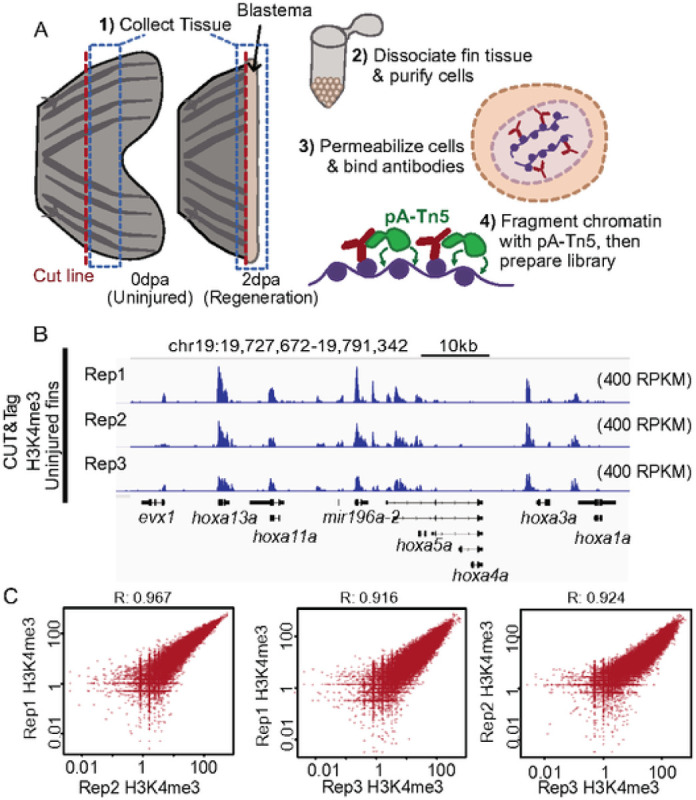
CUT&TAG detects H3K4me3 at the promoter sites of annotated genes in zebrafish caudal fin. (a) The schematic workflow of CUT&TAG applied to zebrafish fins, (b) Genome browser view of H3K4me3 enrichment at select loci. H3K4me3 is detected at the transcription start site (TSS) of three genes with roles in development *(hoxa* gene cluster), (c) Scatter plots displaying the pairwise correlation between the Uninjured (0dpa) H3K4me3 replicates. Correlations are indicative of a Pearson test.

To establish baseline H3K4me3 patterns in adult fins, we performed CUT&Tag on cells harvested from 3 biological replicates of uninjured fins (Fig. 1A). For each replicate, we pooled cells dissociated from 6 uninjured fins, and each pool was divided in half for use in IgG control and H3K4me3 measurements. Similar to prior studies (5), high H3K4me3 levels were detected over gene promoter regions (Fig. 1B). After peak calling (see methods), we identified nearly 49-thousand sites of H3K4me3 enrichment and found there to be a high degree of correlation between replicates (Fig. 1C, S1A), demonstrating great consistency and reproducibility of this technique. Additionally, we observed a high degree of concordance in total CUT&Tag enrichment for H3K4me3 surrounding gene transcription start sites (TSS) (Figure S1B & S1C). These initial results demonstrate CUT&Tag to be reliable and consistent application for the study of epigenetic marks within the heterogeneous mixture of cells that constitute the zebrafish caudal fin (24).

### Measurements of H3K4me3 by CUT&Tag are consistent with prior ChIP-Seq results.

**Figure 2 -:**
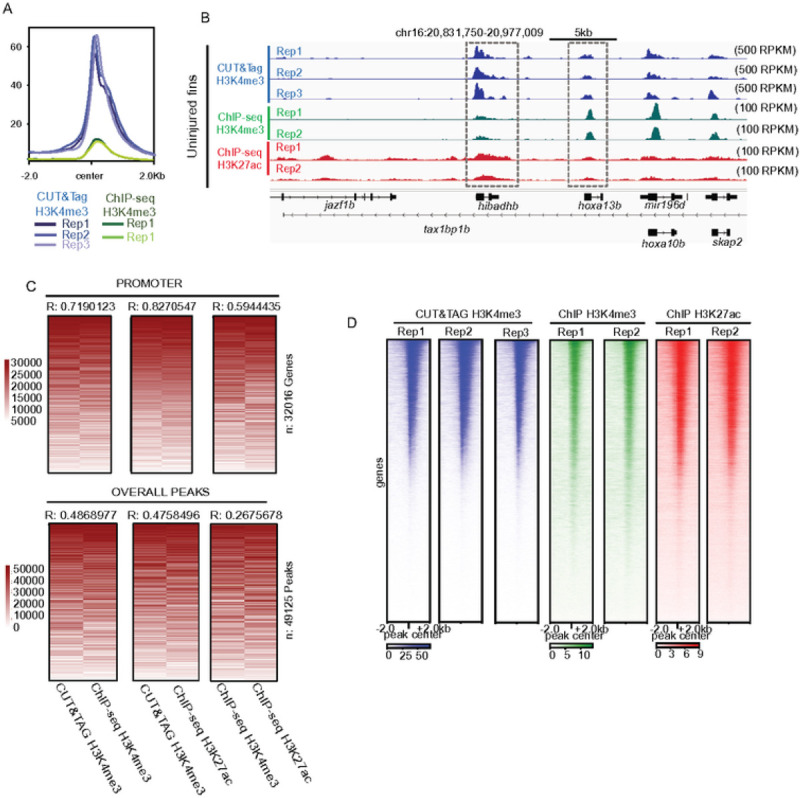
Correlations between H3K4me3 enrichment detected by CUT&TAG and ChlP-seq from the zebrafish caudal fins, (a) Profile plots of H3K4me3 and chromatin modification enrichment at promoter regions in zebrafish fins using CUT&TAG and ChlP-seq. (b) Genome browser view of H3K4me3 CUT&TAG, H3K4me3 ChlP-seq, and H3K27ac ChlP-seq enrichment at select loci. All three signal types are detected at the TSS of *hibadhb* and several *hox* genes, (c) Rank normalized correlation heatmaps between ChlP-Seq and CUT&TAG enrichment over promoters and overall peak regions. Correlations are indicative of a Pearson test, (d) Heatmaps of individual Uninjured (0dpa) replicate data for H3K4me3 from CUT&TAG, H3K4me3 from ChlP-seq, and H3K27ac from ChlP-seq, enrichment at the TSS of annotated genes.

We next compared enrichment of H3K4me3 detected by CUT&Tag with published enrichment measurements acquired by ChIP-Seq. Relative to ChIP-Seq, our CUT&Tag approach detected much higher promoter enrichment scores (RPKM – see methods), demonstrating the improved enrichment signal (as measured by RPKM) (Fig. 2A & 2B). To investigate whether CUT&Tag and ChIP-Seq measurements were similar at enriched loci, we merged replicates, ranked normalized signal independently across promoters or peak regions (to overcome method-specific enrichment differences), and then assessed overall correlations. Measurements at gene promoters were highly correlated when comparing between H3K4me3 CUT&Tag and ChIP-Seq (R = 0.72) (Fig. 2C, 2D, S2A). H3K4me3 CUT&Tag also exhibited high correlation (R = 0.83) with H3K27ac, an another histone modification known to be enriched at actively transcribed genes (25, 26). The observed correlation at promoters was much higher than at peak regions (R = 0.48) or at randomly generated background regions (Fig. 2C), which were uncorrelated (Figure S2B). Overall, these results demonstrate a high degree of consistency across replicates for each method, especially in the context of gene promoters (Fig. 2D).

### Changes in H3K4me3 localization occur during early stages of caudal fin regeneration.

**Figure 3 -:**
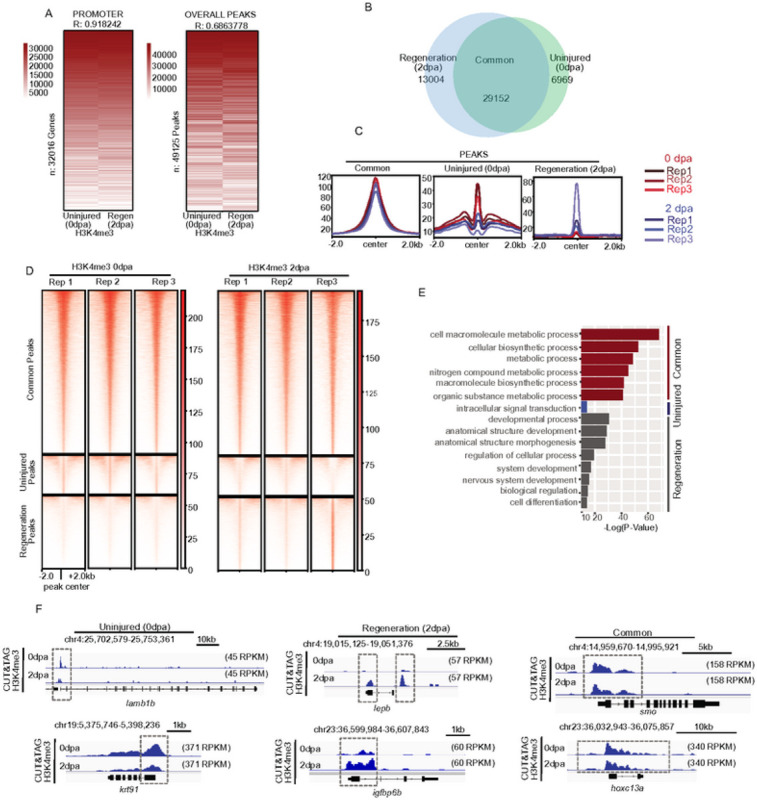
CUT&TAG detects H3K4me3 at the TSS of annotated genes in regenerating (2dpa) amputated zebrafish fin. (a) Heatmap of Pearson correlation values demonstrating significant correlation between uninjured (0dpa) and regenerating fins (2dpa) both within promoters and at peak regions, (b) Venn diagram depicting shared and unique H3K4me3 peaks between Uninjured (0dpa), and Regeneration (2dpa) zebrafish caudal fins samples, (c) Profile plots of H3K4me3 and chromatin accessibility enrichment at classified peak regions (Common, Uninjured (0dpa), and Regeneration (2dpa)). (d) Heat maps of individual Uninjured (0dpa), and Regeneration (2dpa) replicate data for H3K4me3 from CUT&TAG enrichment at regions defined in panel 3B. (e) Gene ontology analysis of classified H3K4me3 marked regions, including Common, Uninjured (0dpa). and Regeneration (2dpa) specific regions, (f) Genome browser view showing enrichment of H3K4me3 at putative regulatory elements for selected genes in Common, Uninjured (0dpa), and Regeneration (2dpa) classes.

Tissue regeneration is achieved by differential expression of a substantial number of genes. To assess regeneration-associated changes in gene promoters, we next applied our CUT&Tag approach to regenerating fin tissues. We collected caudal fins at 2 dpa, a timepoint encompassing blastema formation, which is an essential event of fin regeneration (8), performed CUT&Tag against H3K4me3, and then intersected peaks identified independently for each timepoint. Comparison of H3K4me3 enriched peaks for uninjured (0 dpa) and regenerating (2 dpa) fins identified 29,152 shared peaks present in both samples (Fig. 3A & 3B). Peaks defined as “Common” had consistently elevated H3K4me3 levels across all timepoints and replicates. Peaks defined as “Uninjured” specific had higher H3K4me3 levels across all replicates of 0 dpa, as compared with 2 dpa samples, and peaks defined as “Regeneration” specific had higher H3K4me3 levels across all replicates of 2 dpa samples, as compared with 0 dpa (Fig. 3C & 3D). Interestingly, we found that common and uninjured specific loci were largely associated with binding motifs for FOX and KLF transcription factors, which are well known to have roles in embryonic development (27, 28). Loci classified as regeneration specific were largely associated with motifs for FOS transcription factor, a major component of AP-1 factor which play roles broadly in regenerative context, including zebrafish fins (Figure S3A) (29, 30).

To assess biological pathways associated with H3K4me3 enrichment, we performed the gene ontology (GO) analysis (Fig. 3E) (31). Common peaks tended to reside in close proximity to promoters of genes involved in cell metabolism (Fig. 3F). While uninjured specific peaks generally lacked associations, regeneration specific peaks were associated with embryonic development, morphogenesis, and differentiation (Fig. 3F). For instance, promoters for *igfbp6b* and *lepb* were enriched for H3K4me3 in 2 dpa samples. Interestingly, *lepb* is highly regulated upon fin amputation in zebrafish, and homologs to *igfbp6* are known to be important for regeneration in other systems (10, 32). Additional examples include several genes previously described to have putative roles in fin regeneration (33–36) (Figure S3C). Overall, these analyses provide initial insight into the H3K4me3 changes that occur during zebrafish fin regeneration and highlight locations in the genome where epigenetic alterations occur.

### Changes H3K4me3 levels correspond with moderate changes in chromatin accessibility.

**Figure 4 -:**
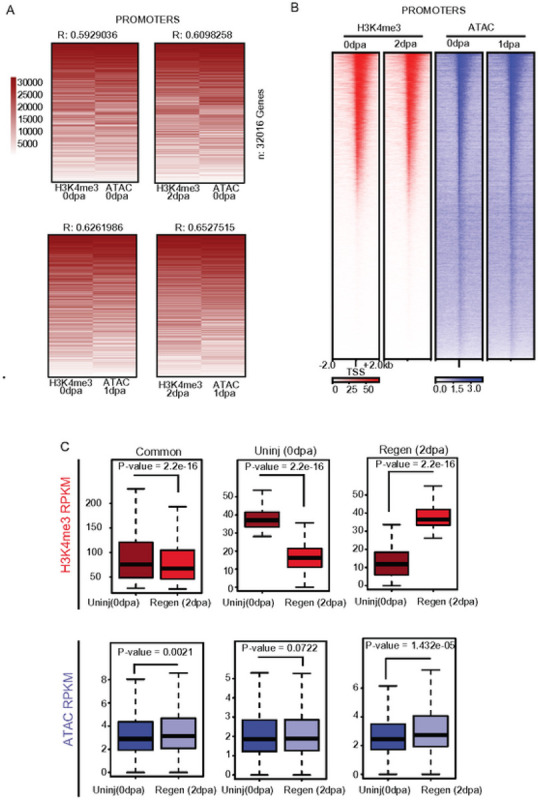
H3K4me3 CUT&TAG correlates with the chromatin accessibility, (a) Heatmap of CUT&TAG and ATAC enrichment (rank normalized) at promoters comparing between uninjured and regenerating tissues. Pearson correlations scores are provided (b) Heatmap of H3K4me3 enrichment and chromatin accessibility enrichment surrounding gene promoters during fin regeneration, (c) Boxplots of H3K4me3 enrichment or chromatin accessibility (RPKM) at defined classes of H3K4me3 marked loci. All P-values are the results of Welch two-sample T-testing

Active gene promoters are often characterized by high levels of H3K4me3 and elevated chromatin accessibility (37, 38), leading us to explore whether changes in chromatin accessibility during the fin regeneration may accompany the observed H3K4me3 changes. To investigate this, we compared enrichment for H3K4me3 at 0 dpa and 2 dpa with previously published chromatin accessibility measurements at 0 dpa and 1 dpa obtained from ATAC-Seq analysis (7, 39). Initial comparisons of H3K4me3 enrichment at gene promoters (Fig. 4B & S4A) indicated a considerable amount of correlation between CUT&Tag and ATAC-Seq signal (Fig. 4A, 4B, S4A), analogous to associations observed in other biological systems (37, 38). We next utilized the previously classified H3K4me3 peaks regions to investigate similar changes in chromatin accessibility, relying on the aforementioned “common” peaks, as well as uninjured specific and regeneration specific loci. As anticipated, regions which gained H3K4me3 between 0 dpa and 2 dpa (classified as regeneration specific peaks) also become significantly more accessible between 0 dpa and 1 dpa (Fig. 4C). Accordingly, regions with lost H3K4me3 during regeneration (classified as uninjured specific) tended to become less accessible (p = 0.072). These results indicate that the majority of already accessible loci (including promoters) remain accessible during fin regeneration, and regions which gain H3K4me3 experienced a moderate but statistically significant increase in chromatin accessibility during regeneration.

### H3K4me3 accumulates during fin regeneration over regions which possessed H3K4me3 in embryos.

Development-related GO terms are enriched in regeneration status samples (Fig. 3E), leading us to hypothesize that changes in H3K4me3 localization during fin regeneration might embody a “return” to embryonic chromatin patterns. To compare regeneration and development samples, we sought embryonic timepoint matching those of 2 dpa regenerating fins. Key transcription factors for appendage development and regeneration include the Msx family of homeodomain-containing transcription factors (40, 41). Upon fin amputation, *msx1b* (*msxB*) is strongly induced in blastema at 2 dpa (40, 41). A previous study reported that *msx1b* is transiently expressed in embryonic fin folds as *msx1b* transcript is uniformly detectable in caudal fin folds at 24 hours post-fertilization (hpf) but restricted to the distal cells at 36 hpf (40, 41). Given the strong and uniform expression pattern of *msx1b* at 24 hpf in caudal fin folds, we chose 24 hpf caudal fin fold as representative fin samples for development.

**Figure 5 -:**
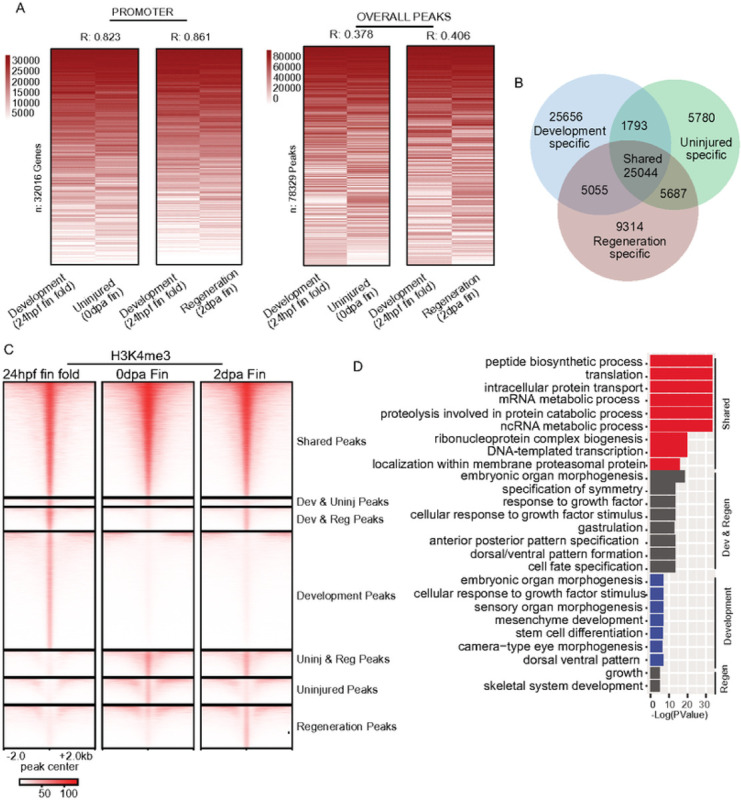
Enrichment for H3K4me3 in embryo fins overlaps with H3K4me3 in regenerating fin samples, (a) Heatmap of enrichment (rank normalized) comparing adult fin and embryonic fin fold samples at promoter regions or overall H3K4me3 peaks. Correlations are indicative of a Pearson test, (b) Venn diagram classifying shared and unique H3K4me3 peaks between Development (24hpf) fin fold samples, and samples from Uninjured (0dpa) or Regeneration (2dpa) zebraflsh caudal fins, (c) Heatmap of H3K4me3 and chromatin accessibility enrichment surrounding peak regions, (d) Gene ontology analysis of classified H3K4me3 peak regions.

We amputated fin folds of ~ 200 embryos at 24 hpf and performed CUT&Tag with IgG and H3K4me3 antibodies. Despite performing measurements on drastically different staged samples, we observed remarkably similar H3K4me3 enrichment patterns at gene promoters in the 24hpf embryonic fin folds compared with regenerating caudal ns (Fig. 5A & S5C). Furthermore, correlation values resulting from comparisons of development and uninjured or regenerating caudal fin samples were only slightly lower (R = 0.82 and R = 0.86, respectively) than values obtained from comparisons between fin timepoints (Fig. 3A, R = 0.92), indicating that H3K4me3 patterns at gene promoters were not drastically different among sample types. This was not the case when we compared H3K4me3 patterns across peaks, which included many intergenic regions. Correlation between development and uninjured or regenerating fin samples was quite modest (R = 0.38 and 0.41, respectively) (Fig. 5A – right), indicating more substantial differences between tissues.

To explore these differences further, we partitioned peak regions with respect to enrichment for each sample type, enabling us to classify peaks as “shared”, when enrichment occurred across all sample types, or “specific”, when enrichment occurred specifically in development, uninjured, or regeneration samples (Fig. 5B & 5C). Remarkably, 35% of regions which acquired H3K4me3 during fin regeneration (5,055 peaks out of 14,369) also possessed H3K4me3 in development (24 hpf embryo samples), as compared with only 24% of regions that lost H3K4me3 (1,793 peaks out of 7,573). In further support of maintained H3K4me3 enrichment over genic loci (as in Figs. 3A & 5A), a relatively large portion of “shared” peaks occurred within gene promoters (21% of peaks). Whereas uninjured- and regeneration-specific peaks tended to occur more frequently over intergenic regions (Fig. 5D). GO analysis revealed that shared peaks were associated with “housekeeping” genes, loci possessing H3K4me3 in both regenerative fins (2 dpa) and in 24 hpf embryos were associated with developmental genes, and no significant ontology terms were identified for H3K4me3 peaks that were lost during fin regeneration (possessing H3K4me3 at 0 dpa but not at 2 dpa) (Fig. 5E). These results support a mechanism in which accumulation of H3K4me3 occurs during caudal fin regeneration over regions which previously possessed H3K4me3 at the earlier developmental timepoints (24hpf), including many developmentally regulated gene promoters.

### Changes H3K4me3 levels at gene promoters are accompanied by gene expression changes.

**Figure 6 -:**
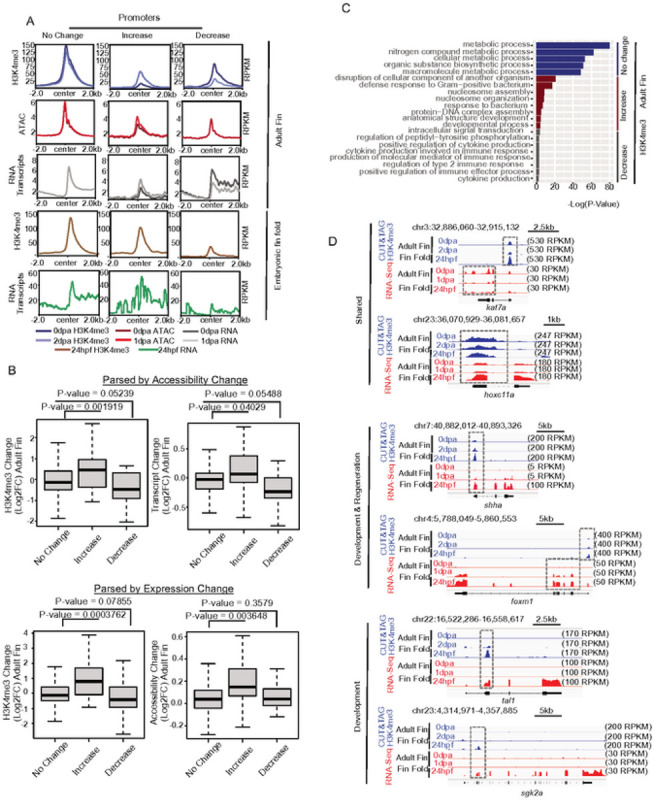
CUT&TAG for H3K4me3 correlates with RNA transcript abundance, (a) Profile plots of merged H3K4me3. chromatin accessibility, and RNA enrichment at gene promoters parsed based on changes in H3K4me3 during regeneration (see methods), (b) Boxplots of H3Mme3. chromatin accessibility, and RNA enrichment at regions defined based on chromatin accessibility changes (top) or gene expression changes (bottom), (c) Gene ontology analysis of regions defined In panel a (d) Genome browser view showing enrichment of H3K4me3 and gene expression (RNA transcript abundance) at the regulatory elements of selected genes All P-values are the results of Welch two-sample T-testing

As noted, high H3K4me3 levels are indicative of gene activation, and loss of H3K4me3 leads to gene expression reduction (38). We therefore investigated whether the observed CUT&Tag H3K4me3 changes during fin regeneration associated with altered gene expression patterns. For this analysis, we first categorized gene promoters based on changes in H3K4me3 levels between 0 dpa and 2 dpa (see methods). Promoters were categorized in a manner similar to our parsing of peak regions, classifying loci as common, uninjured-specific, and regeneration-specific (Figure S6A). In agreement with our prior measurements, chromatin accessibility levels remained mostly stable over promoters during regeneration, and we observed modest but statistically significant increases at 1 dpa for promoters which gained H3K4me3 (regeneration-specific) (Fig. 6A – red profiles & S6B). Changes in RNA transcript levels also followed a pattern highly similar to the observed changes in H3K4me3. Promoters which gained H3K4me3 had higher levels of RNA at 1 dpa compared with 0 dpa, and promoters which lost H3K4me3 experienced a decrease in RNA transcript levels over this same period (Fig. 6A – grey profiles & S6B). Additionally, promoters which acquired H3K4me3 during regeneration also exhibited higher levels of H3K4me3 and a greater abundance of RNA transcripts within 24hpf embryonic fin folds, as compared with promoters that lost H3K4me3 (Fig. 6A – brown and green profiles, respectively & S6C).

To confirm these results, we next parse promoters based on changes in RNA transcript levels, or changes in chromatin accessibility, and then assessed H3K4me3 patterns. For these measurements we again classified promoters using a strategy similar to the one we previously described for H3K4me3 (see methods). Interestingly, H3K4me3 levels increased at promoters which become more accessible, and decreased at loci which lost accessibility (Fig. 6B). In the context of gene expression, we observed a significant increase in H3K4me3 levels at genes which became more transcriptionally active during regeneration (from 0 dpa to 1 dpa) and H3K4me3 significantly decreased at gene promoters which underwent silencing (Fig. 6B). As in our comparisons with 24hpf embryonic fin folds, GO analysis revealed that promoters which maintained or experienced a decrease in H3K4me3 levels were associated with metabolism and housekeeping processes, whereas gene promoters which gained H3K4me3 associated with the developmental processes and establishment of embryonic morphology (Fig. 6C), such as *kat7a* and *hoxc11a* (43, 44). Examples of genes which acquire H3K4me3 during early fin regeneration post amputation and embryonic fin development included *shha* (45, 46) and *foxm1* (47), and examples of genes associated with fin fold-specific H3K4me3 included *tal1* (48) and *sgk2a* (49) (Fig. 6D) (24, 50–53).

## Discussion

Our study demonstrates CUT&Tag to be an effective tool for investigating epigenetic changes during zebrafish caudal fin regeneration. We find there to be a high degree of reproducibility between biological replicates, a strong concordance between CUT&Tag and ChIP-Seq datasets, and a robust agreement with results acquired from RNA-Seq. Furthermore, the relatively few number of cells required for CUT&Tag, the higher signal-to-noise ratio (21), and the feasibility of this technique, as compared with ChIP-Seq, make CUT&Tag particularly amenable to investigations of the adult zebrafish fins. The high degree of sensitivity this technique offers is likely to enable future researchers to assess chromatin changes within discrete cell types, perhaps including purified populations within regenerating tissues (52). Additionally, the feasibility and robustness of CUT&Tag will allow researchers to gain access to more refined timepoints during regeneration, potentially attaining higher resolution of molecular mechanisms underlying the reprogramming process.

Recent technological advances have enabled researchers to characterize numerous tissues at single-cell resolution through measurements of RNA (54) or chromatin accessibility (55). In the very recent past, CUT&Tag methods have been similarly applied (56), and it is therefore conceivable that studies of caudal fin will soon include single-cell epigenetic characterization. It is also likely that improvements in CUT&Tag methods or the closely related CUT&RUN method (57) will allow researchers to investigate changes in transcription factor binding using single-cell approaches (21, 22). Such advances can drastically improve our molecular understanding of the regeneration process, in which numerous epigenetic modifications and transcription factors are known to play critical roles (8, 10, 58).

Our findings revealed a substantial overlap of H3K4me3 localization in 24 hpf embryonic fin folds and 2 dpa regenerating adult fin tissues, providing evidence that genetic and epigenetic programs that are important for embryonic development are repurposed during adult fin regeneration. The regenerative blastema, which forms during 1–2 dpa, is comprised of dedifferentiated cells that arise from a mixture of adult fin tissues, including osteoblasts and fibroblast/mesenchymal cells (9). The mechanisms permitting blastema formation remain poorly understood, but our study raises the interesting possibility that chromatin and epigenetic factors which facilitate development in embryos play important roles in regeneration-based reprogramming processes. So called “bivalent” chromatin modifications reside at developmental genes within embryonic stem cells in a wide range of organisms (59). Bivalent chromatin is characterized by the dual presence of H3K4me3 and H3K27me3 (a silencing histone modification) at gene promoters. This combination of epigenetic marks enables developmental genes to remain silently poised in undifferentiated stem cells, so that they can become rapidly activated during later developmental stages (59). Here we find that one component of bivalent chromatin, H3K4me3, accumulates at developmental genes during the precise timepoint when mature fin cells dedifferentiate to progenitor-like state. Whether H3K4me3 and/or H3K27me3 function as ‘bivalent’ epigenetic factors within regenerative progenitor cells remains unknown and is a compelling topic for future investigation.

It is also interesting to note that cells within the blastema are able to re-use developmental programs/pathways to regenerate fins rather than applying regeneration-specific mechanisms – if such processes exist at all. Markedly, these same developmental pathways are highly conserved in mammals, yet mammals lack the ability to regenerate limbs. It is plausible that an ancestor of mammals maintained these pathways for use in development but lost the ability to reactive them following injury in adults. Like mammals, certain teleost species of cartilaginous and ray fishes like *Cottus gobio* cannot regenerate limbs (60) despite a much closer common ancestor with zebrafish. While it is unknown how divergence among vertebrates occurred, our results indicate that the genes necessary for regeneration are likely present in mammals, but these genes can no longer be activated at the precise time and place for limbs to regrow. It is also worth noting that many mammals are highly regenerative as infants or neonates, but lose the ability to regenerate tissues in adulthood (58, 61, 62). Thus, it is quite conceivable that temporal regulation of chromatin and epigenetic features (as opposed to gene specific mutation or adaptation) are involved in these species-specific limb regeneration mechanisms.

Although the data presented in this study are robust, and we offer an optimistic perspective for the regeneration community, we expect that CUT&Tag technologies will continue to be refined and optimized, and newer adaptations are likely to emerge (22). We anticipate that our data will serve as a useful resource for continued investigation of regeneration-specific chromatin or transcription control mechanisms. With the publication of our study, and the accompanying detailed protocol, it is our hope that CUT&Tag methods will be widely adopted, and the regeneration community will continue to advance as a result.

## Method

### Zebrafish Husbandry and Care

Care and maintenance of zebrafish were conducted in strict compliance with guidelines for animal care and use, securing ethical clearance from the University Committee on Animal Resources at both the University of Rochester Medical Center and the University of Wisconsin School of Medicine and Public Health. The zebrafish were housed and nurtured under conditions that conformed to relevant protocols and ethical standards.

### Harvesting of Fin and Embryonic Tissues

To anesthetize animals for amputation, fishes were submerged in a diluted tricaine solution as per IACUC approved methods. Once immobilized, zebrafish placed one by one on a cutting mat, and their fin tissues were transversally cut at 50% location and carefully transferred to 190ul PBS solution in an Eppendorf tube. For uninjured tissues, fins were cut again at the length expected to be regrown at 2 dpa. Two days after amputation, the regenerated fins were cut for 2 dpa samples. 3 fins per antibody were combined as one sample. After fin amputation, the zebrafish were transferred to a recovery tank for several mi before being returned to their original tanks. For development samples, embryos were cultured in egg water and maintained at 28°C for 24 hours. At 24 hpf, dead embryos were removed, and live embryo were dechorionated using Pronase (Roche,165921) diluted at 2mg/ml final concentration in egg water. Dechorionated embryos were vigorously rinsed multiple times and then moved to a dish containing HBSS (no phenol, no magnesium, no calcium). Embryos were anesthetized with tricaine, and any remaining chorions were removed manually with forceps. Using a curved blade, the fin folds were cut transversally to include a portion of the notochord (see more detail in supplementary protocol). A total of 100 fin folds per antibody were collected into HBSS (no phenol, no magnesium, no calcium) (14, 21).

### Cell Processing and CUT&Tag

The detailed protocol is attached as Supplementary Protocol. The protocol was adopted and modified from previously described methods (14, 21). Uninjured or 2dpa fins were collected in 250μL per 6 fins of cold HBSS (no calcium, no magnesium) in a low-bind microcentrifuge tube. A total of 2–3 fins per antibody were used for each condition. Fins were briefly centrifuged and HBSS was replaced with freshly made digestion buffer (HBSS no calcium, no magnesium, 12.5μM CaCl2, 5mg/mL collagenase type IV (Gibco), and 0.26U/mL Liberase DH (Roche)). A microcentrifuge stir bar (1.5 × 8mm) was placed in each tube, and the tubes were incubated on a stir plate set to 120 rpm in a 35°C incubator. The tubes were either flicked or gently pipetted every 15 min for 45 min – 1 hour.

### Sequencing data

The CUT&Tag libraries from zebrafish fins were pooled and sequenced using services from UW-Biotechnology center on the Illumina NovaSeq 6000 platform. Raw sequencing data generated in this study can be found at NCBI GEO with the accession number (GSE261540). The publicly available RNA data used in this study can be found at NCBI GEO Datasets with accession number GSE146960. The publicly available H3K4me3 & H3K27ac ChIP data used in this study can be found at NCBI BioProject with accession number PRJNA559885. The publicly available ATAC data used in this study can be found at NCBI GEO with accession number GSE146960.

### ChIP and ATAC data analysis

The ChIP and ATAC sequencing data were aligned to the zebrafish genome assembly (GRCz.11, Ensembl release 103) utilizing Bowtie2, followed by conversion to bam format using SAMtools. Unmapped reads were filtered out using samtools, and PCR duplicates were eliminated with picard MarkDuplicates. The H3K4me3 replicate data were merged using UCSC bigwigMerge, and genome browser tracks were generated with deepTools bamCoverage, employing the --normalizeUsing RPKM option for normalization. Peak calling for ChIP data was performed using macs2 bdgpeakcall with the parameters -c 10 -l 100 -g 50. The comparison of peak locations between samples was conducted using Bedtools intersect. For the visualization of ChIP read distribution, deepTools bamCoverage was used to compute normalized read counts in each 100 bp genomic window, with the results visualized in the Integrated Genome Viewer (version 73). The matrix of read counts of all samples was generated and converted by deeptools Multibigwigsummary to the CSV format to be processed in R, enabling us to generate scatterplots and rank-normalized correlation plots.

### RNA data analysis

40–50 fin folds amputated from 24 hpf embryos were pooled for RNA-seq analysis. 24 hpf fin fold RNA-seq analysis was done by Novogene with 40 Million of 150bp paired-end using Novaseq6000. Initial processing steps for RNA-Seq data involved mapping reads to the latest zebrafish genome assembly (GRCz.11, Ensembl release 103) employing STAR-aligner, generating the sorted BAM files. To further identify the relationship between genomic features and gene expression, the matrix of read counts of all samples was generated and converted to the CSV format using deeptool Multibigwigsummary. For visualization of RNA read distribution, deepTools bamCoverage was used to compute normalized read counts in each 100 bp genomic window, with the results visualized in the Integrated Genome Viewer.

### CUT&Tag data analysis

The processing of H3K4me3 CUT&Tag paired-end sequencing reads were aligned to the zebrafish genome assembly (GRCz.11, Ensembl release 103) using Bowtie2. Samtools was employed to filter out unmapped reads, and Picard MarkDuplicates was applied to eliminate PCR duplicates. The H3K4me3 replicate data were then merged using UCSC bigwigMerge, leading to the creation of bigwigs (used for genome browser tracks) through deepTools bamCoverage with the setting --normalizeUsing RPKM. Peak calling was executed with macs2 bdgpeakcall, adopting parameters of -c 30 -l 100 -g 50. The matrix of read counts of all samples was generated using deeptools Multibigwigsummary to generate a CSV format, which was further analyzed using standard tools in R for generation of profile plots, rank-normalized correlation plots, and boxplots. Promoters with increased or decreased H3K4me3 were those with log2FC scores greater than 1 or less than − 1, respectively, as calculated in R from CSV table outputs. For the visualization of the data, deepTools plotHeatmap and plotProfile were utilized. Overlapping peak analysis was conducted using bedtools intersect. Motif identi cation and genomic element percentage piecharts were carried out using the Hypergeometric Optimization of Motif EnRichment (HOMER) software package. Lastly, Gene Ontology Analysis was performed using the ChIP-Seeker R package, leveraging clusterProfiler’s statistical tests for multiple testing correction and setting a significance threshold at 0.05.

## Data Availability

Raw sequencing data generated in this study can be found at NCBI GEO with the accession number GSE261540. The publicly available RNA data used in this study can be found at NCBI GEO Datasets with accession number GSE146960. The publicly available H3K4me3 & H3K27ac ChIP data used in this study can be found at NCBI BioProject with accession number PRJNA559885. The publicly available ATAC data used in this study can be found at NCBI GEO with accession number GSE146960.
